# Extending the mutual information measure to rank inferred literature relationships

**DOI:** 10.1186/1471-2105-5-145

**Published:** 2004-10-07

**Authors:** Jonathan D Wren

**Affiliations:** 1 Advanced Center for Genome Technology, Department of Botany and Microbiology, The University of Oklahoma, 101 David L. Boren Blvd, Rm 2025, Norman, OK, 73019 USA

## Abstract

**Background:**

Within the peer-reviewed literature, associations between two things are not always recognized until commonalities between them become apparent. These commonalities can provide justification for the inference of a new relationship where none was previously known, and are the basis of most observation-based hypothesis formation. It has been shown that the crux of the problem is not finding inferable associations, which are extraordinarily abundant given the scale-free networks that arise from literature-based associations, but determining which ones are informative. The Mutual Information Measure (MIM) is a well-established method to measure how informative an association is, but is limited to direct (i.e. observable) associations.

**Results:**

Herein, we attempt to extend the calculation of mutual information to indirect (i.e. inferable) associations by using the MIM of shared associations. Objects of general research interest (e.g. genes, diseases, phenotypes, drugs, ontology categories) found within MEDLINE are used to create a network of associations for evaluation.

**Conclusions:**

Mutual information calculations can be effectively extended into implied relationships and a significance cutoff estimated from analysis of random word networks. Of the models tested, the shared minimum MIM (MMIM) model is found to correlate best with the observed strength and frequency of known associations. Using three test cases, the MMIM method tends to rank more specific relationships higher than counting the number of shared relationships within a network.

## Background

Most scientific fields are data-intensive, but perhaps even more so for biology and medicine. Sequencing efforts have generated billions of base pairs of genetic information across hundreds of thousands of species, and ushered in the relatively recent completion of the Human Genome Project[1]. Microarrays enable thousands of transcriptional measurements per experiment [2], and high-throughput chemistry enables the simultaneous screening of thousands of molecules at a time for activity[3]. New discoveries among research areas (e.g. genetics, medicine, chemistry) lead to a necessarily increasing amount of specialization as more **objects **(e.g. genes, diseases, phenotypes, chemical compounds, etc.) are discovered to be of research interest. This is reflected by the growth in the number of scholarly journals published every year as well as the number of total records indexed in biomedical literature reference databases such as MEDLINE[4]. In any field, the gain in our cumulative scientific knowledge has the unfortunate effect of narrowing our perspectives as individuals – providing us with far too much information to assimilate, and far too many variables to analyze. Yet the most valuable type of information is often what is not known or apparent to others – information implied by a set of data, facts or associations. History is replete with examples of insights into scientific problems coming from a series of observations from apparently unrelated fields, discoveries or events. But how could one retrieve or compile such information in cases where one is not certain what to look for and the search space is vast? This is the primary reason that methods of data-mining and knowledge discovery are becoming increasingly important in handling this explosion of information.

### Previous research

Most scientific knowledge comes from peer-reviewed articles and is written in free-form text, which is difficult to analyze algorithmically. However, the idea that novel relationships within text could be computationally identified based upon existing relationships has its roots in an approach developed by a researcher named Don Swanson, who used software to identify words shared between article titles [5]. Using their software, called Arrowsmith, Swanson and Smalheiser were able to identify common intermediates between Raynaud's Disease (a circulatory disorder restricting blood-flow to the extremities) and the dietary effects of fish oil, leading to the hypothesis and subsequent proof [6] that compounds within dietary fish oil could alleviate the symptoms of Raynaud's Disease [5,7]. To explain why such a sensible hypothesis had gone unnoticed by researchers in either field alone, the term "non-interactive literatures" was coined. This term, in essence, implies that increasing specialization among all fields results in a relative lack of awareness of the findings in other, less related fields. These entities that do not have known or documented associations, yet share intermediate relationships, have been referred to as "**transitive**", "**implicit**", "**indirect**" or "**inferable**" relationships. Deciding that no relationship exists when no co-mentions exist is somewhat of an over-simplification, but a necessary one. Realistically, several co-mentions between terms could be observed without a definitive relationship present. However, if one uses a greater-than-zero cutoff to define when a relationship exists, false-negatives become a problem: Some co-mentions below the cutoff will constitute a real relationship. Using zero co-mentions as a cutoff is a convenience to avoid this problem even if the end result is that some relationships are declared "known" when they really are not.

While pioneering, a keyword-based method such as Swanson and Smalheiser's is both limiting and highly burdensome, especially where a large body of literature is concerned, because the number of unique keywords grows quickly per record analyzed. Neither is the method amenable to open-ended querying – that is, telling a user what is implicitly related to a query term. Rather, one must essentially begin by postulating a relationship between a query term, A, and another term, C, where a set of intermediate terms, B, can be found that connect the two. Even improvements in visualizing or exploring records that share commonalities and/or define entities of interest [8,9] are limited because they require manual user navigation and analysis of results. Other approaches have attempted to utilize Medical Subheadings (MeSH)[10] or the Unified Medical Language System (UMLS) [11] to engage in open-ended discovery by pairing concepts, counting the number of relationships shared by two terms as a means of judging its implicit significance. However, these approaches do not take into account the fact that the more general the nature of the relationship is, the more connections are likely to be shared by two terms.

It was previously demonstrated that, because the number of associations between terms follows a scale-free, or inverse power-law, distribution, the number of inferable associations with any given term rapidly approaches the maximum number of possible associations as the number of direct associations grows[12]. That is, even if one starts with a term that is only associated with several others, at least one of these is likely to be associated with a very large number of terms. Thus, the starting term will be implicitly associated with most of the network (the "small world" phenomenon). Therefore, the issue is not identifying implicit associations, but somehow judging which of the many implicit associations are worth further examination.

**Figure 1:**
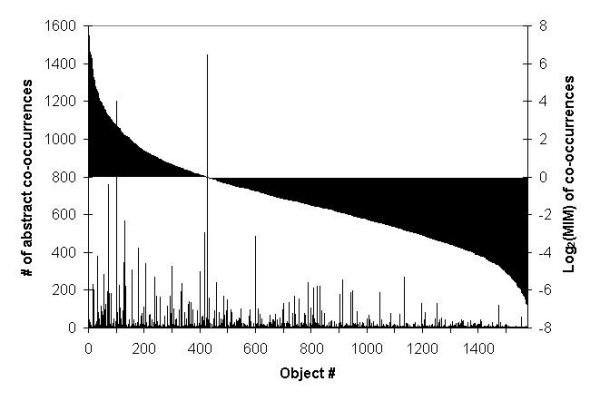
The number of records that two objects co-occur in is loosely correlated with their mutual information. Objects co-occurring in MEDLINE records with "capsaicin" are shown in this graph sorted in descending order by their log_2 _MIM score (see Equation 1), which is plotted on the central axis. The number of records they co-occur within is displayed as vertical bars. Note that a good proportion of objects can co-occur many times, yet at the same time have a negative mutual information measure.

Previous work demonstrated that it was feasible to identify pertinent implicit relationships by ranking inferred relationships and preferentially examining those at the top of the list[12]. One of its shortcomings, though, was that associations between terms are assigned based upon co-occurrence of terms within an abstract. This is a fairly well accepted means of assigning tentative relationships between terms, but when considering the scale-free distribution of objects within the literature, it is apparent that some frequently mentioned objects could be co-mentioned many times with other terms without any actual biological association being implied. Figure 1 uses an analysis of terms related to the term "capsaicin" to illustrate this point. Although the MIM may have drawbacks in identifying broad relationships (for example, see Table 1 – some very pertinent relationships receive modest MIM scores if the terms are common), it is a very straightforward and well-established means of measuring information content between two terms. Such a measure would enable us to pursue more specific relationships – those with high information content. MIM, however, can only be calculated using direct (A-B) or (B-C) relationships rather than implicit (A-C). Thus, the goal here is to test methods of extending the MIM calculation to include implied relationships such that a statement can be made about the **implied mutual information content **of two unrelated terms.

### Identifying literature-based associations

The general approach to associating objects by searching for their co-occurrence within text has been used in many fields as a simple, yet comprehensive way to identify potential associations. In biology and medicine, co-occurrence has been used to identify potential relationships between genes [13,14], proteins [15] and drugs [16]. The disadvantage of this approach is that associations are very general – that is, no specifics on how two objects are related or associated are obtained by this method. False-positives can also be a problem, as terms far apart within the abstract with no apparent association may be included as "relationships". The advantages are that it is easy to implement and comprehensive.

To begin a search for novel, inferable associations within the literature, relevant "objects" of interest in scientific research were first defined by assimilating database entries from relevant databases into one central database. By doing this, both words and phrases can be identified within text, and it permits synonymous terms to be mapped to primary terms. All electronically available literature was then analyzed for associations between objects of interest by searching for their co-occurrence within MEDLINE records (titles & abstracts), summing the total number found. The significance of this collective set of co-occurrences is evaluated using the mutual information measure (MIM), which was originally based upon Shannon's Entropy theory [17], but has also been successful in identifying lexical dependencies [18]. By processing a body of literature that comprehensively covers a topic, field or area, it can be asserted that the current state of knowledge has been approximated, at least on the level of broad object-object associations. All available literature was processed, creating a network of associations for each object. This network can in turn be analyzed for associations shared by two unassociated objects. That is, we can use the network to identify objects that share associations but are not themselves associated. Such objects are said to be *implicitly *associated with each other, and new associations can be potentially inferred by evaluation of their shared associations. Since there are many implicitly associated objects, the relevance of each one is also evaluated using the MIM. However, a MIM can be calculated to evaluate the relevance of an association between A and B and between B and C, but it is not clear how each of these individual scores extends to the inference of an association between A and C. Therefore, we explore and evaluate different methods.

### Methods and algorithms

Code was written in Visual Basic 6.0 (SP5) using ODBC extensions to interface with an SQL-based database, with database queries written in SQL. Programs were executed on a Pentium 4 3.06 GHz machine with 1 GB of RAM and two ultra-fast SCSI hard drives. The National Library of Medicine graciously provided an electronic archive of MEDLINE records in XML format. To obtain a set of common words for analysis, the Merriam-Webster dictionary was parsed into individual words and each word summed by the number of times it was observed within the dictionary. 10,000 words were chosen with dictionary frequencies ranging from 322 to 28. This range was selected so that no extremely common or rare words would be within the list. To create a database of random word associations, only 100,000 titles/abstracts were used. This was done to avoid network saturation (i.e. having a significant number of objects related to every other object) and to ensure that the distribution in the number of associations between words resembled the same power-law distribution observed for biomedical objects.

The occurrence of such objects within scientific text is identified by comparing phrases within MEDLINE records to entries in the object recognition database (ORD). This ORD is built by inputting terms found in several different biomedical databases, all freely available for download. Objects classified as diseases, disorders, syndromes or phenotypes were obtained from Online Mendelian Inheritance in Man (OMIM) [19]; chemical compounds and small molecules were obtained from the Medical Subject Headings (MeSH) database [20]; approved drug names from the Food and Drug Administration; genes were obtained from Locuslink [21], and ontological classifications for genes were obtained from the Gene Ontology consortium [22]. Assimilation of terms is done automatically, but a table within the ORD contains additional biomedical terms to be added or deleted as deemed necessary (e.g., some databases contain vague or uninformative terms such as "survey" or "extended", useless information such as "deleted entry" or errors such as "#NAME?"). Compared to the overall size of the ORD, this table is small (1,007 entries versus over 223,000 terms assimilated) and designed primarily to reduce clutter.

Acronyms for entries, if not explicitly stated within the assimilated database, were obtained from an acronym database[23]. Similarly, spelling variants were also obtained from this database where possible. This database can be accessed online[24]. As an example of spelling variants detected, the user can go to this URL, enter the acronym "ICAM-1" and note the many subtle variations. The acronym resolving heuristic used to construct this database was also used to resolve acronyms within text when they occurred.

### The Mutual Information Measure

A scoring scheme based upon the Mutual Information Measure (MIM) [17] is used to estimate strength of association between co-occurring terms within the literature. It should be noted that other statistical methods of association such as chi-square tests, log-likelihood ratios, z-scores or t-scores could be used as well – these are all means of judging the statistical significance of a relationship. In this paper, however, we will focus on the MIM only as a proof of principle that mutual information calculations can be extended into implicit relationships as well. The MIM has been widely used to quantify dependencies between variables, including co-occurring terms in text [25], and is shown in equation (1):


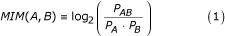


**Figure 2:**
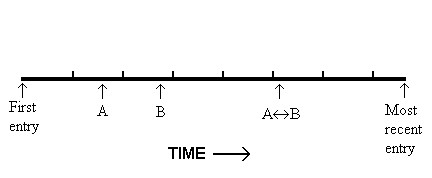
Time-dependency of discovery. All genes, drugs, phenotypes and chemical compounds first appeared at defined times within the literature, even if they were known before then. This prior information is incorporated into mutual information calculations. Shown is a timeline proceeding left to right from the first MEDLINE record indexed to the most recent, with approximate times of first appearance for hypothetical objects A, B and A-B together within a record (title + abstract). Tick marks roughly correspond to 1 million records.

P_AB _is the measured probability that A and B will be observed together in the same abstract, while P_A _and P_B _are the probabilities of observing A or B, respectively, in a given abstract. Furthermore, because scientific research and discovery is a time-dependant process, prior information can be incorporated to refine the probabilities in Equation (1). The describing of a disease or discovery of a gene, for example, will occur at a given point in time (illustrated in Figure 2) within the history of publications. Regardless of an object's overall frequency in the database, the probability it will appear in the literature prior to its discovery is zero. Thus P_A _and P_B _are calculated from their time of first appearance. P_AB _is then calculated using the later of these two dates. P_AB, _P_A _and P_B _are thus calculated as:


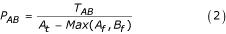



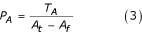



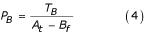


Where T_A _and T_B _are the total number of records A and B are independently mentioned in, respectively, and T_AB _is the total number of records co-mentioning A and B. A_f _and B_f _represent how many records were read in before the first occurrences of A and B were observed, respectively. Max(A_f_, B_f_) represents the larger of the two values between A_f _and B_f_. And A_t _is the total number of records processed.

As an example of how the MIM score is used, assume that the probability A will appear in any given record within a database of records is 10% and the probability of B appearing is the same. If the appearance of A is completely independent of the appearance of B then no information about one can be gained by observing the appearance of the other. The probability both A and B will be observed in the same record is thus 0.1*0.1 = 0.01. The value of *MIM *in Equation 1 then evaluates to 1 and the log value to zero – the information gained on one object by observing the other. If the probability of observing A increases when B is mentioned, then *MIM *> 0. If A and B are rarely mentioned together, then *MIM *< 0. When considering scientific writing style with reference to biomedical objects such as genes, diseases and chemical compounds, there is a probability that two of them might be mentioned together in the same record without having an established association. For example, one of the objects may be very commonly used in many studies (e.g. the gene LacZ is used for staining assays, luciferase is used for luminescence, etc), or one of the objects may be of great scientific/medical interest and authors may make an extra effort to speculate how their results might relate to such objects (e.g. cancer, diabetes, heart disease, apoptosis). The MIM provides a way of quantifying literature-based object dependencies. However, taking the log value can provide a negative weighting to an association when two frequent terms are mentioned together. Optimally, irrelevant or uninformative associations (i.e. those with little mutual information) would be ignored entirely rather than penalized. Therefore, the log function is removed and the equation becomes:


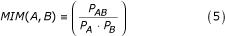


The possibility remains that rare associations might receive a very high MIM score [26], but it is hoped that the fact that many MIM scores are being summed and compared will ameliorate this effect when it occurs.

### Inferring new associations based upon commonalities

**Figure 3:**
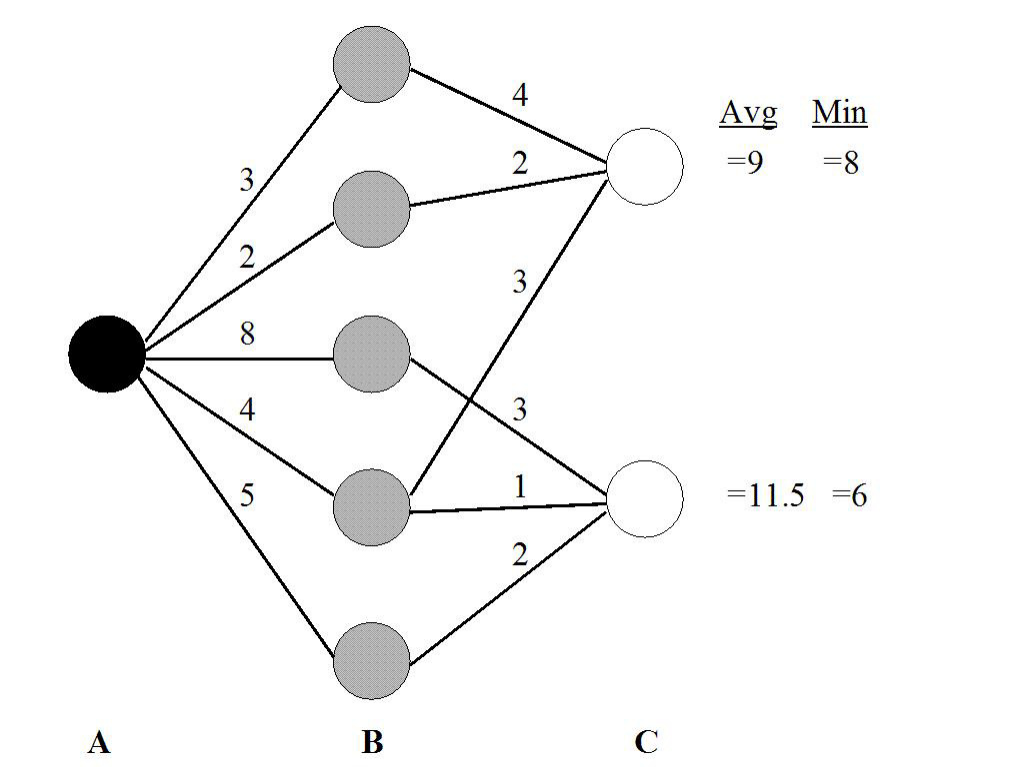
Conceptual illustration of how an inferred association between two objects is identified and evaluated using the methods in Equations 6–7. A primary object of interest (A – black node) co-occurs in records with other objects (B – gray nodes). Each association (A-B and B-C) is assigned a mutual information score, the higher the score the stronger the association between the two. These intermediate associations (B) can then be used to infer an association between A and C (white nodes). Example values are given above the lines. Note that, depending upon whether these values are averaged or the minimum value taken, the rank order of the implicit connections changes. If averaging, the bottom C node receives a higher score (11.5 vs. 9). If taking the minimum, the top node receives a higher score (8 vs. 6).

Figure 3 shows the general conceptual approach undertaken here. Call the primary research object node "A" in a network constructed of MIM scores between objects. For each A there is a set of other objects, or nodes, associated with it by virtue of co-occurrence in the literature. We'll call this set "B" and assuming a total of *t *objects in this set, each individual object can be given the symbol "B_n_", where 0 <*n *<*t*. For each B_n_, there is another set of objects related to it by literature co-occurrence, called "C". Each object in the set C may or may not be connected to the primary object, A. That is, an association may consist of A↔B↔C where an object in the set C also belongs to the set B. The symbol "↔" is used here to represent the existence of a non-directional association between two objects.

Objects in the set C having no literature-based association with the primary object, A, represent associations that have not been previously made, or at least documented, by others. These represent new associations that can potentially be inferred by virtue of their shared associations. Because the number of implicit associations rapidly increases with each established association, the goal here is to provide a quantitative measure of the strength of an implicit association based solely upon the associations shared by two objects. After all, if no known relationship is documented, then these shared associations will be the only way to understand the nature of an relationship between A and C. Since directly associated objects also share associations with other objects, it is reasoned that the strength of known associations can be used to benchmark how well the scores from implicit associations correlate with the relative importance of an association. However, it is not clear how A-C relationships are best evaluated given a set of component A-B_n _and B_n_-C associations. Two models are thus proposed and evaluated, the numeric score obtained by any one of them will only be relevant in terms of how well it assigns a relative importance to each A-C connection within a list.

### Scoring inferred associations

The first model to be tested assumes that the total information content of an implied A-C association can be approximated by the mutual information measure of each component connection. Thus, the MIM scores for each A-B_n _and B_n_-C MIM association is averaged over a total of *t *shared connections and then finally divided by *t *to normalize the total score by the total number of connections. The function for the normalized averaged MIM (AMIM) model is:


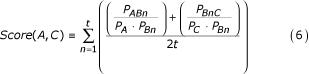


As model by which A-B and B-C values were summed was also considered, but it would be functionally indistinguishable from the AMIM model in terms of ranking implicit relationships, so it was not included.

The second model views the process of inferring an A-C connection as function of each of its component processes, limited in its potential by the mutual information in each step of the inference process. That is, inferring an A-C connection depends upon how much information is in the A-B_n _association as well as the B_n_-C connection, and the information potential an A-C connection will be no greater than the least mutual information given by A-B_n _or B_n_-C. This is equivalent to assuming that a chain can be no stronger than its weakest link. The equation for the normalized minimum MIM (MMIM) model is:


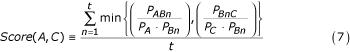


## Results

A total of 12,899,016 MEDLINE records recorded from 1967 to May 2003 were processed in chronological order to create a network of 10,873,926 associations between a total of 112,805 unique objects assimilated from the databases mentioned. When including synonyms, the total number of recognizable phrases for these unique objects was 223,540 (e.g. "IL-6" is a synonym for "Interleukin-6", and the two are treated equivalently).

The distribution of objects found in MEDLINE ranges from more general categories (e.g. "blood", "tumor", "stress", "lesions") that are found in a higher percentage of records ("blood" was the most abundant, being found in 17.5% of all records analyzed) to the more specific. The frequency of objects when plotted follows a power-law distribution and resembles that of a scale-free network, which is reasonable given that new objects are typically studied in terms of their relationship to known objects (law of preferential attachment).

Records were chosen for analysis due to their electronic availability and are also because they are a good source of pertinent information due to their brief, focused nature that presumably contains a summary of the most important findings in each report. Several objects were examined to see if associated objects with high MIM scores correlated with the relative importance of the association. This was done by obtaining summary descriptions of an object from various authoritative sources such as review articles, glossaries or biomedical databases. Table 1 shows an example of associations to an object that were found by scanning all MEDLINE records. Note here that objects with higher MIM scores tend to be objects found in fewer MEDLINE records. Initially this was thought to be problematic because objects highly germane to the biological activity of another object could be down-weighted solely because of their relative abundance. However it was found that when analyzing sets of shared associations in both AMIM and NMIM models, these abundant objects that initially receive low MIM scores subsequently receive much higher scores because they share many high-information content associations with the primary object of analysis, and their cumulative score rises with each one.

Table 1 can be said to reflect the current state of knowledge, as obtainable from scientific abstracts and with reference to biomedically relevant associations to capsaicin. From what is known, a list of what can be inferred is constructed. Each of these secondary associations is used to identify and score implicit relationships as illustrated in Figure 3. As mentioned earlier, a subset of the objects in (C) identified by their associations to the secondary objects (B), will be other secondary objects themselves. That is, they will also be in the set B.

### Model evaluation

**Figure 4:**
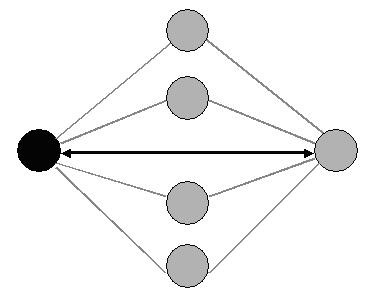
Established associations also share associations with a primary object of interest and can be evaluated purely in terms of their shared associations.

When ranking inferred associations, the goal is for the score assigned to an inference to correlate well with the amount of mutual information gained from any given association. The only reference basis for this is the mutual information contained within established associations. Since they too will frequently contain shared associations, they can be evaluated independently using only their shared associations (Figure 4). Several different methods of ranking inferred associations were evaluated together using the object capsaicin for comparison. Because time must be spent analyzing shared associations to determine the nature of an inferred association, one inference ranking method could be considered superior to another if it yielded a high ratio of relevant to irrelevant associations during the analysis phase. For analysis purposes, the "relevance" of an association will be equivalent to its MIM score – the higher the MIM score, the more relevant the association. Thus, the higher that *known*, relevant associations are ranked within the set of all inferable associations, the better the ranking method is.

To evaluate this, a graph is drawn to reflect the rate that established relationships are discovered within the set of all objects analyzed. The total of all MIM scores for *known *relationships is added together, in order from highest MIM score to lowest, to reflect the fastest rate by which they could be discovered. When plotted, this curve is what would be observed were mutual information preserved exactly (the "exact" curve). Because it's neither expected that all possible relationships are known, nor that mutual information is static as the scientific discovery progresses, it is not anticipated that this curve would or even should be followed exactly (if it were, then that would imply future discoveries could not be more informative than what is already known). However, it is reasonable to expect established relationships with high mutual information content to retain a relatively high mutual information content when evaluated on the basis of its shared relationships. Thus, it is expected that the implicit MIM curve follow the "exact" MIM curve.

**Figure 5:**
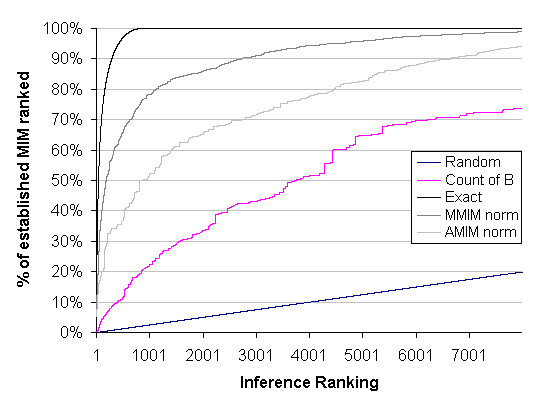
Performance of several approaches to ranking the mutual information contained in inferred relationships for the object "capsaicin"

Figure 5 illustrates what percent of all established associations are identified by each scoring method. The mutual information of associations shared by two objects is ranked by several methods, including the Minimum MIM (MMIM – equation 6) and Averaged MIM (AMIM – equation 7). Objects are ranked here by their shared associations and included in this set are associations that have already been established within MEDLINE as well as those that are implicitly associated. When an established association is encountered within this ranked list, its MIM is added as a percentage of the sum of all MIM scores. When all established associations have been ranked by each method, the total will add to 100%. The faster an inference ranking method approaches 100%, the better it scores objects with high mutual information. Shown for comparison is what the curve would look like if each established association were ranked in the exact order of its highest to lowest MIM scores ("Exact"). Also shown is how quickly established associations would be found by guessing at random ("Random"), and how quickly established associations would be found when counting the number of intermediates ("Count of B").

To gain a better quantitative estimate of performance, 50 objects were chosen at random from both the MEDLINE and random word databases. Each object was analyzed to identify and rank other objects that shared relationships with it as described and the area under the curve (AUC) was taken for each of the ranking methods shown in Figure 5. For the MEDLINE network, the average AUC for the MMIM was 43% ± 9%, for the AMIM it was 42% ± 8%, and using the count of shared relationships was 9% ± 7%. The difference between the MMIM and AMIM was not large (p < 0.29 using a 2-tailed paired t-test) but was slightly biased by a relatively few examples where AMIM performed very well. Out of the 50 trials, MMIM performed better 35 times, equally 11 times and worse 4 times. What is most pertinent is that both MIM methods ranked objects with high mutual information content significantly higher (p < .000001) than counting the number of shared relationships.

A peculiar effect was noted with the average MIM-based scoring model: Some implicitly associated objects received higher MIM scores than the primary object itself, which is also analyzed as a control. There tend to be relatively few, sometimes none, such instances per analysis, but it occurs when a relatively rare object shares several or more associations with the primary object. This effect was not present in the minimum MIM model.

### Using a random word network to estimate significance intervals

The scores assigned by inference methods so far have no meaning by themselves, but only as a means of ranking the potential relevance of an inference. Because the majority of database objects will be present in the list of implicit connections, the question naturally arises as to where a significance cutoff can be drawn. A range of significance for a given MIM score can be estimated by analysis of a random word network in which we would expect that meaningful relationships are only encountered by chance.

**Figure 6:**
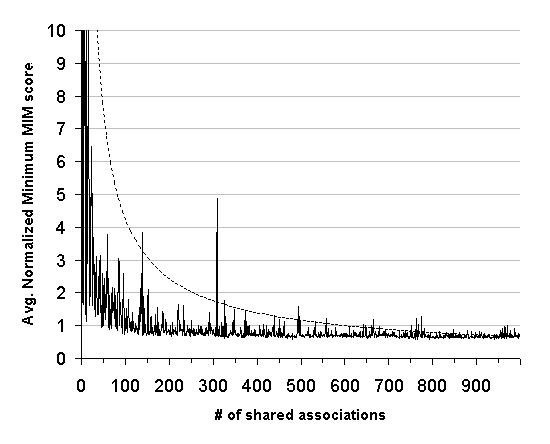
The average normalized minimum MIM (ANMMIM) score trends towards a value slightly less than one when a network of random words is analyzed for the relevance of implicit associations. The dashed trend line is a power-law fit to the standard deviation of the ANMMIM.

Since the MMIM model performed slightly better than the AMIM model, we evaluated it using the random words database. Words in the random network were effectively chosen at random from the Merriam-Webster dictionary (see Methods and algorithms), and so relevant associations between these words co-occurring within MEDLINE records should occur predominantly by chance. An uninformative association (i.e. chance alone could explain the number of term co-occurrences) would have an average MIM score of 1 (e.g. see Equation 5). Thus, summing a set of *t *random associations and dividing by t would also be expected to have an average (normalized) MIM score of 1. This is true for any set of A-B associations as well as a corresponding set of B-C associations, thus an average value of 1 should still be obtained when calculating the average minimum MIM score. Figure 6 shows a plot of the average minimum MIM score (with standard deviation) by the number of shared associations of words in a random network. The average minimum MIM score trends towards a value below 1 (average value from 500 to 1000 shared connections = 0.7), which is not surprising given the nature of writing: It is not random, so two words would not necessarily co-occur together with a probability that is proportional to their relative frequencies. This also suggests that a log value of zero for a MIM score may not be the most effective dividing line between informative and non-informative associations. The values obtained from this analysis provide us with a way of estimating a significance cutoff for implied mutual information analysis. As Figure 6 also shows, the fewer shared associations between two objects, the higher the average normalized MMIM score is as well as its standard deviation.

### Evaluating capsaicin

Using the example of capsaicin brought up earlier, we analyzed it using the methods described to identify and rank objects sharing relationships with it (Table 2). When ranked by counting the number of shared relationships, the more general relationships (as mentioned in Table 1) tend to rank towards the top, such as calcium and neurons. This seems a good means of identifying general relationships, but each of the objects on this list is hardly specific to capsaicin. Ranking by MMIM, however, changes the nature of the types of objects that are ranked highly to those that share molecular/physiological relationships with capsaicin by their effects upon nerves and transmission of impulses. For example, the **ileum **is frequently used to test capsaicin effects because of its contractile response. **Tachykinin**s **Substance P **and **Neurokinin A **as well as the neurotransmitter **acetylcholine**[27] are responsible for afferent nerve transmission in response to capsaicin, the response to which can be blocked by antagonists such as **tetrodotoxin**[28] or **atropine**[29]. These implicit objects share relationships with B objects of all different types mentioned in the methods & algorithms section, but the ones that tend to score highest are the ones that share several highly informative relationships with the A object. In general, these informative relationships tend to be objects that are mentioned much more frequently with the A object than any other object within the literature. Acetylcholine, for example, is associated with many neurological processes, but has relatively high MIM scores with other objects related to capsaicin such as bradykinin, atropine, neuropeptide Y and substance P, which are all molecules that affect the transduction of sensory signals.

### Re-evaluating Swanson's original discoveries

Finally, we also sought to re-evaluate some of Swanson's original hypotheses as has been done in other text-mining studies [10,11,30]. It makes less sense, however, to attempt to judge performance based upon whether or not Swanson's studies or hypotheses could be replicated, *per se*. To do so presumes that Swanson's initial study was the "correct" way of finding relevant implicit relationships and Swanson did not employ the open-discovery model in these examples anyway. It would be useful to know, however, where Swanson's predictions rank among others using models in which implicit relevance is judged by counting the number of shared relationships and where it is evaluated by the MMIM. Both Raynaud's and Migraine headaches were analyzed as starting objects (A), the goal being to find all C objects that share relationships and rank them by their relevance. Both known and implicit relationships were displayed. The top 10 results are summarized in Table 3, and the entire dataset is available by request.

When ranking implicit relationships by the number of shared relationships, fish oil scored #1025 in the Raynaud's list and magnesium (the link Swanson found with migraines [31]) scored #166 in the Migraine list. When ranked by MIM, fish oil scored #1512 and magnesium was ranked #458, lower in both cases. The scores for Raynaud's Syndrome<->Fish oil were lower than expected. Upon examination, Swanson's discovery of this link, although validated experimentally [6], has apparently not generated a lot of continued experimental research interest in this area in the 15 years since then. A search via Ovid on "(raynaud or raynauds or raynaud's) and (eicosapentaenoic or docosahexaenoic or fish oil)" yielded only 5 papers, three of which were text mining papers including Swanson's original study [5,30,32], the fourth was the 1989 validation study [6] and the fifth was a study showing that fish oil did not have a significant effect upon Raynaud's phenomenon in mixed cryoglobulinemia (a syndrome in which Raynaud's is one of many symptoms)[33].

Examining the relationships that tend to rank highly in both models it is apparent that, when ranking by the number of shared relationships, the higher-scoring entries tend to be more general and vague in nature (e.g., links to "blood", "development", "females" and "males"). When ranked by the MMIM, their relevance to the object in question is more readily apparent. For example, **sumatriptan **is a drug used to treat migraines and other items ranking highly on the list such as **nausea**, **vomiting**, and **dizziness **typically accompany migraines. Notably, one of the important links that Swanson used to surmise the role of magnesium is also on this list: **Seizures**, which cause migraines.

## Discussion

Information retrieval (IR) methods are limited to querying what is known; yet often the most valuable information is what is not directly known. Mutual information measures have been used successfully in many IR applications, and a method has been presented here to extend it to inferable associations. We find that the normalized MMIM method of ranking inferences based upon their shared associations correlates best the level of currently established mutual information. A good correlation is suggestive that mutual information is being captured even though evaluation proceeds indirectly, through intermediates. For simplicity, we have used a cutoff of zero co-occurrences to suggest that no association between objects has been made, but it is quite possible that a number of co-occurrences could be noted between two objects yet no specific relationship between them documented. Or additionally, a certain relationship may be known between the two, but other important relationships still remain to be inferred. At this point, however, it is not clear how this would effectively and quantitatively be taken into account.

The method reported was applied to biomedical research, but could ostensibly be applied to any domain in which the goal is to identify undiscovered relationships. Importantly, this method of automated inference ranking provides a quantitative way of prioritizing inferred associations when available literature is growing rapidly in size and scope.

## Figures and Tables

**Table 1:** An example of co-occurring objects found in MEDLINE with a primary object of interest, capsaicin. A brief summary of what capsaicin is shown at top, with database objects of associated biomedical research interest in **bold**. Below are the MIM scores for each of these co-occurring objects, along with their relative rank in the list when sorted by score. A total of 2,069 objects co-occurred with capsaicin in the body of literature analyzed. *Capsaicin *is the active compound in chili peppers that causes their burning sensation. It acts upon a small family of **capsaicin receptors**, which have been found in sensory and vagal **neurons**, and allows a **calcium **influx into these cells causing them to fire and send heat-related signals to the CNS. Capsaicin can cause **neurogenic inflammation **upon application, and in high enough concentrations it is a **neurotoxin**.

**Primary Object (A)**	**MIM Score**	**Secondary Objects (B)**	**Relative rank**	**# of records containing B**
Capsaicin	0.24	Calcium	1132	303,041
Capsaicin	10.42	Neurotoxin	66	7,612
Capsaicin	35.33	Neurogenic Inflammation	20	2,258
Capsaicin	89.62	Capsaicin Receptor	11	914
Capsaicin	0.96	Neurons	509	589,031

**Table 2:** Analysis of objects that share relationships with capsaicin, ranked both by the number of relationships they share and by their minimum mutual information measure (MMIM). Frequency (Freq) is the number of co-mentions the two objects share in the literature.

**Ranked by # of shared relationships**						**Ranked by Minimum MIM**			
			
**Query term (A)**	**Implicit relationship (C)**	**Shared rels**	**MMIM Score**	**Freq.**			**Implicit relationship (C)**	**Shared rels**	**MMIM Score**	**Freq.**
			
capsaicin	Capsaicin	844	2623	-			Capsaicin	844	2623	-
capsaicin	development	800	135	250			Tachykinin	348	595	351
capsaicin	Neurons	776	217	1835			Atropine	601	564	327
capsaicin	Brain	769	174	118			Substance P	676	495	1513
capsaicin	membrane	769	172	194			Tetrodotoxin	456	448	246
capsaicin	Secretion	759	219	260			Neurokinin A	279	440	229
capsaicin	Skin	749	157	591			Acetylcholine	681	436	167
capsaicin	intracellular	746	224	158			Neuropeptide Y	640	417	536
capsaicin	Calcium	743	189	190			Hexamethonium	312	392	130
capsaicin	nervous system	743	189	138			Ileum	578	383	119

**Table 3:** Swanson's implicit discoveries analyzed either by counting the number of shared relationships or by the MMIM score. Frequency (Freq) is the number of co-mentions the two objects share in the literature. Notice that the *type *of relationship that ranks higher with the MMIM tends to be more specific and informative, while the terms that share the most relationships tend to be more general and vague.

**Ranked by # of shared relationships**						**Ranked by Minimum MIM**			
**Query term (A)**	**Implicit relationship (C)**	**Shared rels**	**MMIM Score**	**Freq.**		** Implicit relationship (C)**	**Shared rels**	**MMIM Score**	**Freq.**

migraine	Migraine	1442	4149	-		migraine	1442	4149	-
migraine	development	1362	216	348		Headache	1140	1519	4032
migraine	Lesions	1291	264	164		Sumatriptan	270	1038	1152
migraine	nervous system	1280	311	227		Nausea	899	886	323
migraine	pathogenesis	1253	375	475		Vomiting	889	732	242
migraine	Muscle	1251	201	184		Dizziness	696	669	136
migraine	Blood	1236	63	272		Ataxia	674	562	62
migraine	Tumor	1236	93	158		Vertigo	467	511	208
migraine	Females	1223	243	200		Somnolence	389	497	19
migraine	Males	1219	238	169		Seizures	1057	493	198
**Query term (A)**	**Implicit relationship (C)**	**Shared rels**	**MMIM Score**	**Freq.**		** Implicit relationship (C)**	**Shared rels**	**MMIM Score**	**Freq.**
Raynaud's	Raynaud's syndrome	200	2088	-		Raynaud's syndrome	200	2088	-
Raynaud's	development	198	45	11		Raynaud's phenomenon	183	1204	45
Raynaud's	Artery	195	86	36		Raynaud's disease	118	936	30
Raynaud's	Muscle	193	53	2		mixed connective tissue disease	121	870	13
Raynaud's	pathogenesis	192	127	18		Scleroderma	161	862	48
Raynaud's	Lesions	192	71	26		connective tissue diseases	151	842	31
Raynaud's	Vessels	191	108	10		Arthralgia	133	761	6
Raynaud's	Blood	190	16	26		overlap syndrome	81	619	1
Raynaud's	Skin	190	24	18		Calcinosis	104	592	7
Raynaud's	Cutaneous	189	200	13		progressive systemic sclerosis	124	587	14
